# Developments in molecular epidemiology of aging

**DOI:** 10.1042/ETLS20180173

**Published:** 2019-06-20

**Authors:** Sara Hägg, Daniel W. Belsky, Alan A. Cohen

**Affiliations:** 1 Department of Medical Epidemiology and Biostatistics, Karolinska Institutet, Stockholm, Sweden; 2 Department of Epidemiology & Butler Aging Center, Columbia University Mailman School of Public Health, New York, U.S.A.; 3 Department of Family Medicine, University of Sherbrooke, Sherbrooke, Canada

**Keywords:** aging, biomarkers, methods, molecular epidemiology

## Abstract

The field of molecular epidemiology of aging involves the application of molecular methods to measure aging processes and their genetic determinants in human cohorts. Over the last decade, the field has undergone rapid progress with a dramatic increase in the number of papers published. The aim of this review is to give an overview of the research field, with a specific focus on new developments, opportunities, and challenges.

Aging occurs at multiple hierarchical levels. There is increasing consensus that aging-related changes at the molecular level cause declines in physiological integrity, functional capacity, and ultimately lifespan. Molecular epidemiology studies seek to quantify this process. Telomere length, composite scores integrating clinical biomarkers, and omics clocks are among the most well-studied metrics in molecular epidemiology studies.

New developments in the field include bigger data and hypothesis-free analysis together with new modes of collaborations in interdisciplinary teams and open access norms around data sharing. Key challenges facing the field are the lack of a gold standard by which to evaluate molecular measures of aging, inconsistency in which metrics of aging are measured and analyzed across studies, and a need for more longitudinal data necessary to observe change over time.

## What is molecular epidemiology?

The research field *molecular epidemiology* is a young, quickly developing field. It is described as the intersection of two different research areas, molecular biology and epidemiology. The word *molecular* refers to small components such as proteins, metabolites, as well as interactions among them. Molecular epidemiology consists of the application of molecular methods in cohort settings, e.g. proteomics and metabolomics, to conduct statistical analysis using an epidemiologist's toolbox on new types of biological data. Traditionally, epidemiological research was done on registers linking different data sources together in order to associate exposures with disease risk, but without any molecular or biological information included. Adding molecular data allows epidemiologists to investigate pathways and underlying biology explaining links from exposure to disease. Although the term *molecular epidemiology* was coined as early as 1973 [1], an increase in the number of papers published on the topic was not seen until 2009, going from a few hundred per year to several thousand per year. Today, advances in high-throughput molecular methods, declines in costs of molecular assays, and fast expansion of computational power are driving a rapid pace of development in the field. Hence, the aim of this review is to give an overview of the sub-field of molecular epidemiology of aging, with a specific focus on new developments, opportunities, and challenges.

## What is aging and how is it measured?

To study the molecular epidemiology of aging, it is first necessary to have an idea of what aging is, beyond the passage of time. Unlike the molecular epidemiology of cancer, which could link molecular markers to clinical diagnoses or pathology-defined cancer sub-types [2,3], there is no clinical or pathology-lab definition of aging. Nonetheless, the evidence for aging as a process in itself appears incontrovertible: each species has a typical lifespan with some variation, and different species can often show parallel aging processes at very different timescales. For example, mice and humans age relatively similarly, but at timescales roughly 40-fold different, with no known mutations in either species that could come close to bridging that gap. These lifespans are relatively constant even in different conditions and with different causes of death: hunter–gatherers can live well into their 80s and, generally, do not die of the chronic diseases that affect modern populations [4].

Aging is obviously linked to chronic diseases, but it is important to distinguish the two. Many chronic diseases, notably cancer and cardiovascular disease, are the end result of processes that can start relatively early in life and do not necessarily occur in all individuals. Thus, even while the presence of these diseases is related to chronological age, it is not clear whether they share an etiology with biological aging.

A growing body of science agrees that aging represents a set of biological processes that can be studied. However, there is not yet a consensus as to what those biological process are. What consensus does exist suggests that aging is multifactorial [5–7]. The accumulation of wear and tear and loss of regenerative capacity are among the mechanisms. But questions remain about the universality of these mechanisms; for example, a range of species across the tree of life appear not to age at all [8], at least not at evolutionarily relevant timescales [9]. Thus, a biological understanding of what we know about aging is crucial for the interpretation of emerging molecular metrics and approaches.

**Figure 1.**
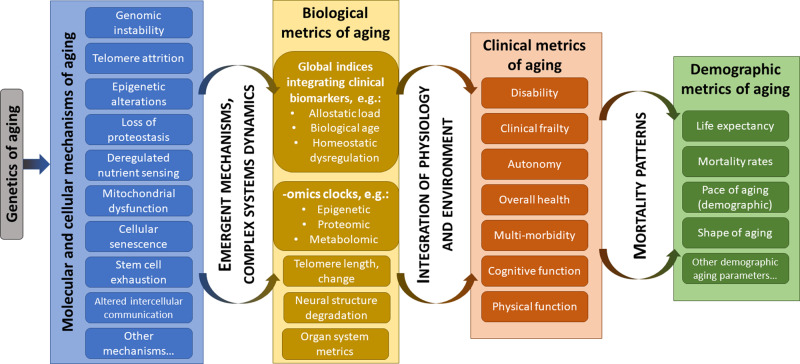
A multi-level view of aging and its metrics. Molecular and cellular mechanisms are drawn from López-Otín et al. [7] for illustration and are not intended to represent an exhaustive or definitive list. Reliable, simple metrics of these mechanisms applicable in an epidemiological context are generally not yet available.

Aging occurs at multiple hierarchical levels, from molecular changes at the cellular level to changes in organismal physiology to changes in organismal function [10]. Aging can also be studied as changes in population-level demographics [11]. Because these levels represent complex systems, aging at a higher level of organization partly represents an emergent property of structure at lower levels. In this context, it is appropriate to consider what we know about aging at different hierarchical levels and how this might inform ways to measure it in molecular epidemiology (Figure 1).

### Molecular and cellular mechanisms of aging

Like most of biology, aging reflects the interplay between genetic and environmental influences. Cross-species variation in lifespan suggests a genetic component. In humans, studies of relatives estimate the heritability of lifespan, or the fraction of variation related to genetics, to range from 10 to 30% [12]. Molecular genetic studies suggest that there may be differences between the genetics influencing the normal range of lifespan variation and genetics influencing extreme longevity [13], although there is also evidence for some commonality [14]. The role of the inherited genome in determining variation in human lifespan remains a subject of active study.

At the molecular/cellular level, seven to nine key ‘pillars’ or ‘hallmarks’ of aging identified in recent reviews include genomic instability, telomere attrition, epigenetic alterations, loss of proteostasis, deregulated nutrient sensing, mitochondrial dysfunction, cellular senescence, stem cell exhaustion, and altered intercellular communication [5,7]. However, the authors acknowledge that these classifications are tentative, that there is substantial variation across species, and that there is still marked uncertainty about the causal roles in aging and links between these mechanisms. And some major mechanisms/theories (e.g. immunosenescence [15,16]) are hardly mentioned. The science is ongoing, and in several years, the list is likely to change, as it did for the hallmarks of cancer [17,18].

From a molecular epidemiology perspective, the pillars/hallmarks can serve as a general guide to processes that should be measured [19,20]. However, many pillars require costly or invasive measurements, for example, the senescence-associated secretory phenotype [21] is difficult to measure in accessible tissues like blood, and biomarkers are not available for all processes [19,20]. Moreover, each of the pillars/hallmarks is itself multifactorial [7], arguing against one-to-one correspondence between biomarkers and aging processes.

### Metrics of biological aging

At present, molecular measures of aging in wide use are mostly not direct measures of the pillars/hallmarks. The exception is leukocyte telomere length, a biomarker of cellular senescence [22]. However, telomere length is contested as a biomarker of human aging [23]. As measurement technologies develop, it may be feasible to integrate pillars/hallmarks of aging into molecular epidemiology. For now, most epidemiologically feasible metrics of aging biology reflect global organismal decline. A common feature of these metrics is that they integrate information across an array of biological markers to produce a generalized score. These metrics can broadly be divided into two classes: high-dimensional ‘clocks’ based on omics data (DNA methylation, gene expression, metabolomics, proteomics, etc.) and those relying on a smaller set of standard clinical biomarkers measured from blood chemistries, hematology, anthropometry, and organ function tests such as blood pressure and spirometry.

The best-known omics clocks combine information from sets of DNA methylation marks. Referred to as ‘epigenetic clocks’, these measures estimate a biological age equivalent to the chronological age at which the observed methylation status would be approximately normal in the general population [24]. Although clocks based on transcriptomic, metabolomics, and proteomic data have been proposed (see Jylhävä et al. [25] for a review), they have yet to be taken up by many other studies. In general, clocks are generated via machine learning approaches and produce biological age estimates that are highly similar to subjects' chronological ages, suggesting high precision. But these clocks are not necessarily good predictors of health states, although recent clocks specifically developed to measure health states show stronger relationships with morbidity and mortality [26,27]. Because epigenetic changes are among the pillars/hallmarks of aging, it is tempting to see the epigenetic clocks as being closely related to cellular and molecular mechanisms of aging. While this is possible, the nature of the algorithms used to generate the clocks renders them something of a black box. The mechanism or mechanisms that make(s) the clocks tick remain(s) unknown.

Clinical biomarker metrics are typically less well correlated with chronological age as compared with the epigenetic clocks. However, in studies comparing different measures of biological aging, the clinical biomarker metrics are more strongly associated with health status, mortality, and also with risk exposures linked to shorter healthy lifespan [28–31]. Some of the best-known examples of clinical biomarker composite metrics include: (A) allostatic load, designed to quantify the effect of chronic stress on physiological decline [32,33]; (B) the biological frailty index, which indicates a percentage of biomarkers for which a subject is at risk [34]; (C) various metrics of biological age, which quantify system integrity at a cross-section by matching an individual's physiology to a reference based on chronological age or risk of mortality, e.g. [35,36]; (D) homeostatic dysregulation, measured as a statistical distance or regression tree representing how aberrant an individual's physiology is relative to a reference norm, typically the sample mean [37]; and (E) the pace of aging, designed to quantify the rate of decline in system integrity across multiple organ systems jointly [38].

Beyond these global metrics of aging and related processes, there are also metrics that reflect processes at different hierarchical levels, such as in organs and organ systems. For example, the Framingham Risk Score might be seen as a proxy for cardiovascular aging, though it also incorporates behavioral and lifestyle factors (e.g. smoking). In addition, various approaches to measure age-related processes in the brain — molecular, structural, and functional data acquisition — may help to quantify brain aging [39] and are sometimes linked with all-cause mortality [40]. Some of these indices were developed for clinical risk prediction rather than a measurement of aging *per se*. A challenge in the development of biological aging measures from clinical data is that the distinction between aging and disease is unclear. Even though many of the metrics used in clinics likely reflect aspects of aging at intermediate levels of biological organization, it is beyond the scope of this paper to discuss them further.

Each of these methods has strengths and weaknesses, and few comparative studies have been conducted. Few such studies conducted to date suggest that biological aging measures derived from the same type of data tend to capture similar, but not identical information, whereas biological aging measures derived from different types of data show very little overlap. For example, in one recent study, estimates of biological aging from different epigenetic clocks correlated with one another in the range of Pearson *r* = 0.3–0.5, whereas correlations between epigenetic clocks and physiology-derived measures of biological aging tended to fall below *r* = 0.1 [28]. These results, together with similar findings from other studies [30,31], suggest that no single measure is sufficient to quantify all aspects of biological aging. Organismal aging is a highly multivariate process. The future may be in integrative analysis of multiple measures. The increasing availability of a wide variety of methods provides growing opportunities for researchers wishing to quantify various aspects of aging.

### Clinical and demographic metrics of aging

The emerging methods to quantify biological aging described above complement an already existing set of measures used in clinical settings to measure aging-related decline. These measures focus less on precise quantification of biological processes of aging [41] and more on decisions related to patient care [42]. There are numerous measures of disability [43], clinical frailty [44,45], autonomy [46], overall health [47], and multi-morbidity/chronic disease burden [48], not to mention domain-specific measures of function such as cognition [49] and physical function [50], each with many sub-domains. Progress in the field also requires the development of new phenotypic measures that can be used to anchor the biological data. Such measures are under development, notably measures of facial aging [51] and patient-reported outcomes. An important step in the further development of quantifications of biological aging is establishing how such new aging measures relate to an aging-related decline in phenotypic aging, notably physical and psychological functions [28,52].

Research is also needed to evaluate how measures of biological aging relate to demographic measures of aging, such as population-level life expectancy, mortality rate, or pace and shape of mortality curves [53]. These and other metrics allow comparisons of aging across populations or species. Studies are needed to test if population differences in demographic measures of aging are reflected in differences in biological aging among individuals within populations [54].

## New developments in molecular epidemiology of aging

### Bigger data, bigger analysis: from hypothesis-driven discovery to hypothesis-free data mining

Datasets in molecular epidemiology have shifted from a focus on individual molecules of interest to comprehensive surveys of biological substrates, e.g. whole-genome single-nucleotide polymorphism (SNP), DNA methylation, and gene expression data. To drink from the big data firehose [55], analytic strategies in molecular epidemiology have shifted from hypothesis-driven targeted testing to hypothesis-free data mining. For example, gene discovery research has shifted from testing candidate genes suggested by animal studies to conducting genome-wide association studies (GWAS) that survey millions of genetic variants across the genome. This approach has yielded replicable discoveries for hundreds of traits, especially as sample sizes have grown from thousands of individuals to tens and hundreds of thousands of individuals [56]. But, discovery in aging research remains in the early stages. Recent GWAS have changed approaches from comparisons of extremely long-lived persons with controls in the normal age range [57,58] to studies of parental lifespan [14,59,60], which allow much larger samples. And new methods are being applied to conduct GWAS of longevity processes in samples of still-living individuals [61]. The candidate genes *APOE* and *FOXO3A* are the best-studied longevity genes, and they have replicated in GWAS of multiple designs (reviewed here [62]). Meanwhile, the same type of study design is now seen for projects in epigenetics, where population-based cohorts with similar genome-wide DNA methylation data join forces to identify methylation marks associated with a trait of interest. Aging studies on the epigenome have been far more successful; thousands of DNA methylation changes have been associated with the aging process in humans and validated across different cohorts [63–65]. Another type of study using recent GWAS findings within genetic and molecular epidemiology is the application of Mendelian randomizations to assess causal associations in aging. Several papers have used this design to provide evidence for causality using genetically predicted telomere length on disease outcomes [66–68]. The benefit of conducting studies using genotype information is that biases such as reverse causation and confounding, which are problematic for other biomarkers or observational associations, are avoided. An additional approach to hypothesis-free data mining is machine learning analysis. Investigators use algorithms to cycle through large numbers of potential models and pick the one that best fits the data. This best-fitting model can then be tested in a separate dataset to establish its predictive value. There is a range of machine learning analyses in use in molecular epidemiology. For example, lasso regression analysis of GWAS data is being used to develop prediction algorithms [69], although these have not yet been applied to studies of aging-related phenotypes; elastic-net regression analysis of epigenomic data is being used to develop novel measurements of the aging rate [24]; and neural-net analysis of a range of data types, including transcriptomic data, is being used to develop aging biomarkers and identify drug targets [70]. As machine learning approaches continue to mature, studies will be needed to compare methods and define best practices for implementation.

### Bigger collaboration: high-dimensional interdisciplinarity and team science

Collaborations in molecular epidemiology have changed in two important ways. First, the increase in the size of molecular datasets and the complexity of analysis have driven increases in research team interdisciplinarity. Epidemiologists collaborate with biochemists and geneticists, statisticians and computer scientists, and clinicians and ethicists to generate and analyze giant databases. In parallel to this increase in interdisciplinary collaboration, the size of research teams has also increased. The scale and complexity of big data molecular epidemiology had driven a shift in discovery science from research within individual laboratories to research pursued through teams of laboratories organized into global consortia. To effectively mine molecular datasets with tens of thousands to millions of measurements, very large sample sizes are needed. To meet this challenge, investigators and funders have formed global consortia and invested in massive collaborative research endeavors to achieve samples of tens or hundreds of thousands. In aging research, the CHARGE (Cohorts for Heart and Aging Research in Genomic Epidemiology) Consortium, which has conducted a large epigenome-wide association study (EWAS) on educational attainment including epigenetic clock analyses [71], as alluded to above, is an example.

### Bigger access: open-access data and open-source analytic tools

An important outcome of the large-scale public investments in molecular datasets is the emergence of new data-sharing policies and norms. Funders and high-impact journals have implemented data-sharing requirements, forcing investigators to make their data available within the research community through online databases. Although the actual accessibility of molecular epidemiology data is constantly debated [72,73], many databases with open-access data are available, including the U.S. National Institutes of Health-operated database of Genotypes and Phenotypes (dbGaP) (https://www.ncbi.nlm.nih.gov/gap) and Gene Expression Omnibus (GEO) (https://www.ncbi.nlm.nih.gov/geo/). The Nature-hosted journal *Scientific Data* publishes descriptions on open datasets and provides a list of recommended repositories for each data type [74]. Other resources that have emerged are large population-based cohort collections open freely for academic use. The most prominent example is the UK Biobank, where half a million individuals from the UK have been sampled, interviewed, and linked to electronic health records [75]. Currently, 1380 research applications are approved for using UK Biobank data, 115 of them are listed as age-related. Another important open resource for epidemiological studies in aging is the U.S.-based Health and Retirement Study (HRS) [76]. The HRS is a longitudinal study of 20 000 individuals aged 55–64 years with repeated assessments on cognitive domains and a multitude of biological data (e.g. genomics, epigenomics, and telomeres). Many more cohorts are also open for use, some with restrictions on collaborations (e.g. the Womens Health Initiative (WHI)), but still accessible. Funding bodies investing in cohort collections are often firm in their demands for data sharing, such as the National Institute on Health (NIH), and administer databases to facilitate these services.

Alongside changing norms about data access, norms about access to tools with which to analyze data have also changed. Driven in part by the integration of computer scientists and bioinformaticians in big data molecular epidemiology, habits from the computational field with source code sharing and open-source developments of new tools and statistical packages are spreading. GitHub (https://github.com/) is an example where researchers openly share scripts for processing and analyzing data. Bioconductor within the R-project is another example where software packages for cleaning, performing statistical analyses, and plotting data are published in peer-reviewed journals and freely available for use [77]. There are also many web-based tools for analyzing data, e.g. the Human Ageing Genomic Resources (HAGR, http://genomics.senescence.info) [78]. Perhaps, the most impactful example in molecular epidemiology of aging is Steve Horvath's online calculator (https://dnamage.genetics.ucla.edu/new) for processing DNA methylation data and outputting predicted values of epigenetic clocks, blood cell counts, and a host of other parameters [79]. Because the calculator was fast and easy to use, it was taken up immediately by dozens of research teams, instantly harmonizing epigenetic clock measurements across dozens of studies and catalyzing a flood of research testing association between the clocks and age-related traits in many cohorts [24]. Because of the standardized values across cohorts, results were comparable between studies, and a large-scale meta-analysis of clock associations with mortality was achieved only a few years after the first clocks were published [80].

### Challenges

New developments in the molecular epidemiology of aging have generated new opportunities, but many challenges remain. Foremost among challenges in molecular epidemiology of aging is the lack of a gold standard measurement of aging itself, as noted above. In animal studies, the standard metric of aging is lifespan; genes and therapies can be screened for effects on how long an animal survives. But this metric is challenging to implement in human studies, not least because humans live for a long time. Longevity nevertheless remains a standard outcome in molecular epidemiology. Case–control studies compare exceptionally long-lived centenarians to controls of typical older age. Prospective studies test the prediction of mortality over follow-up periods of 5 or 10 years. But because they depend on observing variation in survival, these studies can only examine the end of the human lifespan. They have little power to tackle the lifetime of exposures occurring before study follow-up begins, and how these factors may influence the biological aging process. And because follow-up begins in later life, the fastest aging members of the population have often died already or become too ill to participate. Studies of healthy lifespan, or ‘healthspan’, face similar challenges; to study the onset of age-related disease, follow-up must begin relatively late in life. For example, the TAME Trial, which is planned as the first large-scale randomized trial of drug therapy to modify aging, will recruit subjects aged 65–79 years [81]. Thus, molecular epidemiology studies of human lifespan and healthspan struggle to study the life-course process of aging.

Several new methods aim to address this challenge by quantifying processes of biological aging in real time, as alluded to above in the description of different clocks. More recently, the second generation of epigenetic clocks have been developed using two-stage designs in which a first stage develops an algorithm to capture age-patterning of physiological parameters and a second stage fits methylation data to the first-stage algorithm values [26,27]. These clocks are better predictors of morbidity and mortality than first-generation clocks, opening opportunities for applications in etiological and intervention studies. They also suggest a direction for the application of methylation and other omics data in the study of aging, and provide guidance on how to train future models without using chronological age as a time scale [82].

A second challenge facing molecular epidemiology of aging is the lack of longitudinal data on proposed molecular measures of aging. Aging is, at its essence, a process of change over time. Longitudinal repeated-measures data are, therefore, essential to its measurement [83]. A critical test of any proposed measure of aging is that a faster rate of change must forecast morbidity, disability, and mortality. However, because of limited availability of longitudinal data, this test is rarely conducted. Instead, studies more often compare older individuals to younger ones and attribute differences to aging-related changes. This cross-sectional approach confounds an aging-related change with cohort effects (i.e. differences in exposure histories of individuals born at different times). Moreover, because disability and mortality risk accelerate with advancing chronological age, older research participants represent a more selected population than younger ones. Specifically, those able to participate in research at advanced ages are relatively long-lived as compared with their birth cohorts.

An example of the limitations of cross-sectional data in aging biomarker analysis comes from research on telomeres. Telomere studies relying on cross-sectional data established associations of shorter telomere length with morbidity and mortality [22]. But longitudinal studies testing associations of faster telomere erosion over time with similar endpoints have produced more equivocal data [84], undermining confidence in telomeres as a biomarker of aging in molecular epidemiology research. Longitudinal epigenetic clock studies are yielding a similar pattern of results. Analysis confirms that epigenetic clocks ‘tick’, i.e. they change over time as expected [85]. But the variation in the rate of change in epigenetic clocks is not systematically associated with other indications of accelerated aging [28] and no data yet link differences in the rate of epigenetic aging with differences in lifespan or healthspan. Such longitudinal evidence is necessary to advance the translation of aging biomarkers, especially for clinical research and patient care, where the objective is to deliver therapy that slows the rate of aging. A promising example comes from research using physiological data to quantify biological aging. Studies of physiology-based measures of biological aging link a faster change with a decline in physical and cognitive functioning [38,86] and suggest that intervention to slow aging may slow the rate of decline in physiological integrity [87]. Longitudinal data are needed in molecular epidemiology to test the validity of ‘rate-of-change’ measures of aging based on molecular data to establish utility for clinical applications.

### Future directions

There are several priorities for research to advance molecular epidemiology of aging, both in terms of data and resources, as well as regarding metrics of aging. First, as noted above, the development of longitudinal data resources to track biological measures of aging over time is needed. Follow-up of life-course longitudinal studies and integration of new molecular measures should be a priority for researchers and funders. In parallel, the generation of repeated-measures molecular data within clinical studies can shed light on potential to slow or reverse biological changes occurring with aging.

Second, the continued development of platforms and resources to facilitate open data sharing is needed to maximize the impact of investments in data generation. This is happening in the GWAS field with online repositories of summary statistics (e.g. http://atlas.ctglab.nl/) and interactive analytical platforms to use these summary statistics to investigate trait heritability, genetic correlation, and to conduct Mendelian randomization analysis (http://ldsc.broadinstitute.org/ldhub/, http://www.mrbase.org/). As large-scale methylation, gene expression, and other molecular datasets become available, tools for open analysis of these resources will be needed. In parallel, open access analytical methods to compute algorithm-based measures of biological aging, as are available for the epigenetic clocks, can help advance the field.

**Figure 2.**
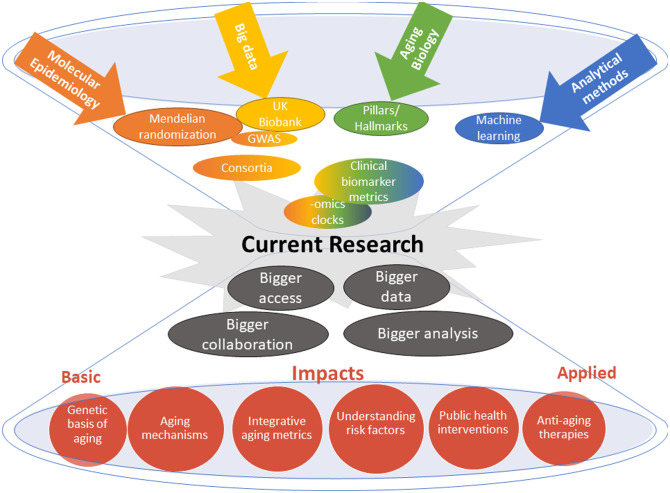
An overview of current research practice in the molecular epidemiology of aging. The field is rapidly advancing from traditional hypothesis testing to hypothesis-free research aims using big cohorts, together with new modes of collaborations and data practices. The graph intends to summarize past, present, and future directions in the field where researchers are approaching from different angles working together to move the research front forward.

Third, given the current development in the field of molecular epidemiology of aging (summarized in Figure 2), researchers have to find alternative ways for rewarding junior scientists as the team science advance with large-scale collaborations and sometimes hundreds of authors on a single paper. Adapting the traditional way of publishing, as has been done in the GWAS field with shared authorship positions, may be a new common practice also in aging research to be able to run the large efforts needed.

Regarding the advancements of aging metrics and models, different efforts are wanted. First, new research is needed to link emerging molecular measures of aging with hypothesized molecular mediators of aging processes. Current data suggest that different measures of aging correspond to different underlying biological processes, but which measures correspond to which processes remains mostly unknown.

Second, new models are needed to integrate multiple measures of aging into patient-level profiles. Existing methods combine information from multiple methylation marks, gene transcripts, or clinical biomarkers. Ultimately, the integration of measures of aging from across these substrates could lead to better predictions of individual aging trajectories.

Third, consensus endpoints are needed for studies aiming to develop and validate novel measures of aging. These should include change with advancing chronological age and the prediction of measures of both healthspan and lifespan. Taken together, the field of molecular epidemiology of aging is quickly moving forward, leading to exiting new developments with great possibilities for breakthrough research and clinical implementations in the following years to come.

## Summary

- Aging is multifactorial and can be viewed at different levels (molecular, biological, clinical, and demographic).
- Today, there is no consensus on which metrics best capture the individual aging process and what models should be used to fit the data.
- The field of molecular epidemiology of aging is shifting from traditional hypothesis-driven analysis of specific markers to hypothesis-free discovery research using datasets that aim to comprehensively measure different biological substrates. This shift is changing the type of data on which the field focuses, the tools and methods used to analyze those data, and the nature of research collaborations.

## Abbreviations

GWAS: genome-wide association studies
HRS: health and retirement study
NIH: National Institute on Health

## Appendix. Glossary of terms

Molecular epidemiology of aging: The study of aging within the intersection of two different research areas: molecular biology and epidemiology. It is the application of molecular methods to generate biological data in cohort settings with statistical analysis using an epidemiologist's toolbox.
Metrics of aging: Measurements of biological processes associated with aging at multiple hierarchical levels, from molecular changes at the cellular level to changes in organismal physiology and function, as well as on demographics of aging.
Aging clocks: A type of aging metrics constructed from omics-based data that ‘tick’, i.e. change over time. The best-known clocks combine sets of DNA methylation marks, referred to as ‘epigenetic clocks’, estimating a biological age equivalent to chronological age in the general population.
Hallmarks/pillars of aging: At the molecular/cellular level, key factors of aging include genomic instability, telomere attrition, epigenetic alterations, loss of proteostasis, deregulated nutrient sensing, mitochondrial dysfunction, cellular senescence, stem cell exhaustion, and altered intercellular communication (Kennedy et al. [5]; López-Otín et al. [7]).
Healthspan: The healthy part of the lifespan, i.e. the number of years with good health in life.
Pace of aging: Quantification of the rate of decline in system integrity across multiple organ systems. Longitudinal repeated-measures data of aging metrics are essential for assessing change over time
